# Lysosomal storage disorder in non-immunological hydrops fetalis (NIHF) - more common than assumed? Report of four cases with transient NIHF and a review of the literature

**DOI:** 10.1186/1750-1172-7-86

**Published:** 2012-11-08

**Authors:** Catharina Whybra, Eugen Mengel, Alexandra Russo, Franz Bahlmann, Christoph Kampmann, Michael Beck, Elke Eich, Eva Mildenberger

**Affiliations:** 1 Department of Neonatology, University Medical Center of the Johannes Gutenberg University Mainz, Mainz, Germany; 2 Department of Lysosomal Storage Disorder, Villa metabolica, University Medical Center of the Johannes Gutenberg University Mainz, Mainz, Germany; 3 Department of Pediatric Oncology, University Medical Center of the Johannes Gutenberg University, Frankfurt, Germany; 4 Department of Obstetrics and Gynaecology, Buergerhospital Frankfurt am Main, Frankfurt am Main, Germany; 5 Department of Pediatric Cardiology, University Medical Center of the Johannes Gutenberg University Mainz, Mainz, Germany; 6 Department of Neuropediatrics, Frankfurt Hoechst, Germany

**Keywords:** Non-immunological hydrops fetalis, Lysosomal storage disease, Transient hydrops, Congenital ascites, Clinical approach

## Abstract

**Background:**

Lysosomal storage disorders (LSD) are a rare cause of non immunological hydrops fetalis (NIHF) and congenital ascites. The reported incidence is about 1%. The incidence of idiopathic NIHF is estimated to be about 18%.

**Patients and methods:**

We report four cases with transient hydrops fetalis resulting from LSD and performed a literature review on LSD with NIHF and congenital ascites in combination.

**Results:**

At present, 12 different LSDs are described to be associated with NIHF or congenital ascites. Most patients had a family history of NIHF, where the preceding sibling had not been examined. A diagnostic approach to the fetus with NIHF due to suspected LSD either in utero or postnatal is suggested. Transient forms of NIHF and/or ascites in association with MPS IVA, MPS VII and NPC are described for the first time in this publication.

**Conclusions:**

LSD should be considered in transient hydrops. Enzymatic studies in chorionic villous sample or amniotic cultured cells, once the most common conditions associated with fetal ascites or hydrops have been ruled out, are important. This paper emphasizes the fact that LSD is significantly higher than the estimated 1% in previous studies, which is important for genetic counseling as there is a high risk of recurrence and the availability of enzyme replacement therapy for an increasing number of LSD.

## Background

Hydrops fetalis is a serious fetal condition defined as an abnormal accumulation of fluid in two or more fetal compartments, including ascites, pleural and/or pericardial effusion, and skin edema. In some patients, it may also be associated with polyhydramnion and placental edema. Isolated congenital ascites is usually considered as part of the clinical picture of hydrops fetalis, in which the severity of peripheral edema is of a mild degree and ascites is the dominant clinical sign [1,2]. Hydrops fetalis has been a well-recognized fetal and neonatal condition throughout history. Before routine immunization of Rh-negative mothers in the 1970s, most cases of immunological hydrops were due to erythroblastosis from Rh alloimmunization. More recent recognition of factors other than isoimmune hemolytic disease that can cause or be associated with hydrops fetalis led to the use of the term non-immunological hydrops fetalis (NIHF). Currently, NIHF (reported incidence of 1 in 2000–3000 pregnancies [3]) is more common, comprising 85-90% of all described cases [4,5]. The precise incidence of hydrops fetalis is difficult to elucidate, because many cases are not detected prior to intrauterine fetal death and some cases may resolve spontaneously in utero. Although the incidence is very low, NIHF accounts for a disproportionate share (3%) of overall mortality in the perinatal period [6-8].

Inborn errors of metabolism (IEM) may cause hydrops fetalis*.* Most of these are lysosomal storage diseases. Today, around 14 different lysosomal storage diseases (LSD) have been reported as being associated with NIHF and congenital ascites [9-11]. The cause of the accumulation of excessive fluid within the peritoneal cavity in infants with LSD is a source of considerable controversy in the literature [12]. The mechanism contributing to the development of hydrops fetalis in storage diseases may involve the obstruction of venous blood return resulting from visceromegaly secondary to accumulation of storage material [13]. Anemia may be a trigger, being caused by either hypersplenism or the reduction of erythropoietic stem cells caused by infiltrating storage cells. Other conditions that may trigger ascites in LSD are congestive heart failure, hypoproteinaemia and liver dysfunction [12].

In this paper we present four cases with transient NIHF and a literature review on the incidence of LSD in NIHF. A diagnostic approach to the patient with NIHF or ascites due to suspected LSD either in utero or postnatally is established.

The purpose of this work is to report four cases, which have in common a transient hydrops fetalis, normal appearance in the postnatal period and progressive deterioration with time. All of them were diagnosised as LSD. Until now transient hydrops fetalis in association with LSD has been barely reported [14,15]. A mortality rate of 100% of IEM in association with a NIHF is assumed [16], however, transient NIHF with a good perinatal outcome has hardly been described.

## Case reports

### Case I

In a term male neonate of non-consanguineous parents with two healthy siblings (birth weight 3250 g, length 48 cm, head circumference 36 cm, Apgar Score 9/9/10, umbilical artery pH 7,24), prenatal ultrasound revealed hydrops fetalis with ascites at as early as 24 weeks of gestation. Prenatal infection was ruled out in fetal blood and any immunological causes were excluded. Amniocentesis showed a normal male karyotype. After birth he was breathing spontaneously. He had a normal phenotypic appearance, bilateral inguinal hernia and a mild hepatosplenomegaly, but no detectable ascites or pleural effusion. Echocardiography and ophthalmological examination were normal. Neonatal cholestasis and icterus praecox with elevated direct bilirubin was assessed. Hepato-IDA-scintigraphy showed patent extrahepatic biliary duct. Liver biopsy revealed foamy cells typically seen in Niemann Pick disease. Enzymatic measurements showed normal values for sphingomyelinase, ß-glucosidase, sialidase and acid lipase, excluding Niemann Pick A disease (NPA), Gaucher disease and Wolman disease. Variant Filipin staining led to the misdiagnosis of NPC. Neonatal cholestasis was treated with ursodeoxycholic acid*,* and then resolved spontaneously. Although the patient showed nearly normal neurological development (slight language development retardation), mild joint contractures and bilateral avascular necrosis of the femoral head, NPC as the slowly progressive form was suspected. Eight years later, the patient was presented in our clinic for a trial of substrate reduction therapy of NPC. The patient had no elevation of chitotriosidase and no horizontal saccades typical for NPC. In contrast, eye examination revealed corneal clouding. In addition, hip dysplasia was seen. Detection of the deficiency of ß-glucuronidase (0,0069 mU/ml) finally yielded the diagnosis of MPS VII.

### Case II

**Figure 1:**
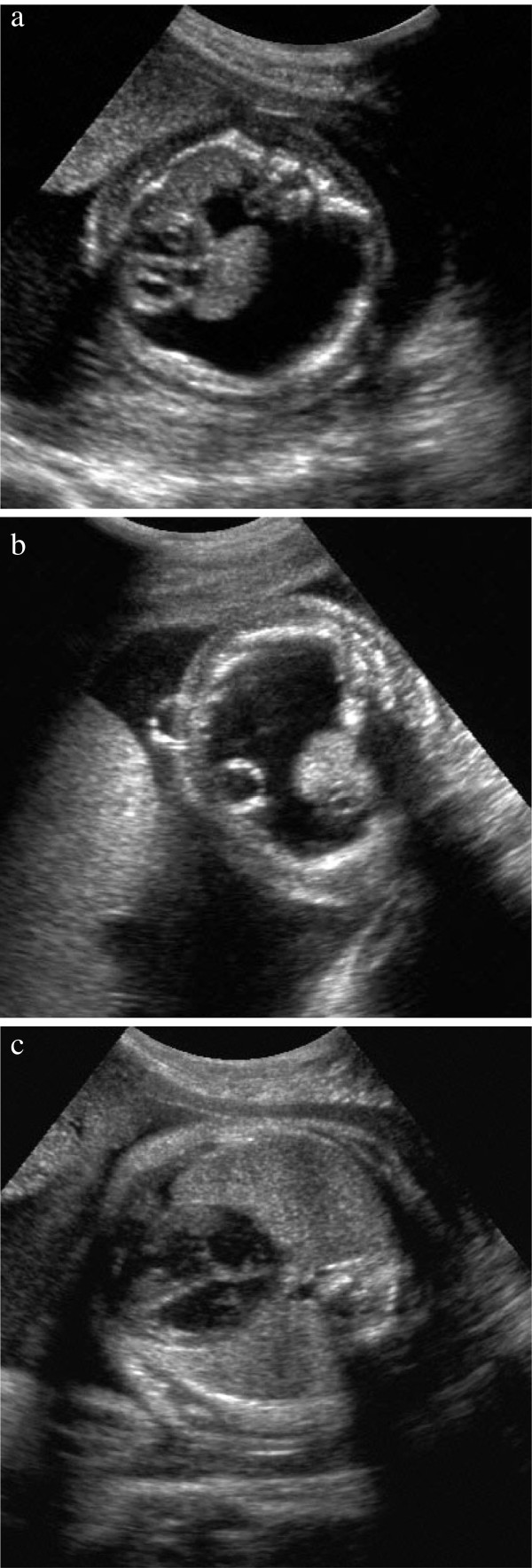
**a: 22 weeks of gestation and massive hydrothorax in case II. b**: 24 weeks of gestation, punction of hydrothorax and insertion of a thoraco-amniotic shunt in case II. Correct placement of the intrauterine shunt system. **c**: Regression of the hydrothorax at 31 weeks of gestation in case II.

The first child of non-consanguineous parents presented with hydrops fetalis, in particular massive hydrothorax (Figure 1a) at 22 weeks of gestation. A drainage of the intra-thoracal fluid was established by ultrasound-guided foeto-amniotic shunting at 24 weeks of gestation (Figure 1b). Thereafter, the female fetus developed normally (Figure 1c) and was born by Caesarian section at 39 weeks of gestation (birth weight 3560 g, length 52 cm, head circumference 35 cm, Apgar Score 9/10/10, umbilical artery pH 7,34). 24 hours after birth the neonate of normal phenotype was admitted to the neonatology ward due to bradycardia and acrocyanosis. There was no evidence of infection. Echocardiography and ECG were normal. Soon after, she was discharged home. At age three years she showed toddling gait and weakness of musculus quadriceps femoris, as well as beginning kyphoscoliosis. N-acetyl-glucosamin-6-sulfat sulfatase deficiency with a residual activity of 0,141 nmol/mg/17 h (normal 12-26 nmol/mg/17 h) confirmed the diagnosis of MPS IVA. Mutation Analysis revealed GALNS c.463 G → A in Exon 5 and Intron 13 (IVS13-1 G → A).

### Case III

In this case, the parents were consanguineous. The mother had had two previous pregnancies with spontaneous abortion. In the third pregnancy the fetus presented with intrauterine hydrops fetalis. The male preterm was born at 29 weeks of gestation by Caesarian sectio because of premature labour (birth weight 1550 g, length 41 cm, head circumference 27,5 cm, umbilical artery pH 7, 12). His neonatal period was uncomplicated. He could walk at age 20 months and showed minor mental retardation. At age one and a half years he developed a kyphoscoliosis. The diagnosis of MPS IVA by detection of N-acetyl-glucosamin-6-sulfat sulfatase deficiency with a residual activity of 0.234 nmol/mg/17 h ( normal 12–26 nmol/mg/17 h) was only made four years later due to continuing disproportionate growth retardation (length far below 3^rd^ percentile) and skeletal abnormalities (thoracolumbar gibbus, ulnar deviation).

### Case IV

The male fetus presented with intrauterine ascites at 30 weeks of gestation. Prenatal infection was ruled out in fetal blood and any immunological causes were excluded. Amniocentesis showed a normal male karyotyp. The male neonate, born at 36 weeks of gestation presented with slight ascites, which resolved during the first weeks. Neonatal jaundice was prolonged for eight weeks and was accompanied by mild cholestatic liver enzyme elevation. He had a normal phenotypic appearance. After one year, hepatosplenomegaly was found in the routine examination of the child. The chitotriosidase was elevated (2504 nmol/ml/h, normal range 20–100 nmol/ml/h). Sphingomyelinase, ß-Glucosidase and ß-Glucuronidase were normal. Subsequently, NPC was suspected. Bone marrow punction revealed storage cells typical for NPC. Positive Filipin staining ensured the diagnosis. RFLP (restriction fragment length polymorphism) showed mutations in the NPC1 Gene.

## Review of the literature

A systematic review in PubMed of the pertinent literature, especially including those reported cases or case series with LSD associated with NIHF and congenital ascites, was carried out. Studies on prenatal ultrasonography, extensive pre- and postnatal investigations and postmortem pathological examination were taken into consideration. No date limit was set.

## Results and discussion

Bellini et al. reviewed 225 relevant articles on non-immune hydrops fetalis which described 6,361 individuals. All 6,361 patients were sub-classified into one of the following 14 diagnostic categories listed in Table 1[17].

**Table 1:** **Causes of non-immunological hydrops fetalis and their relative frequency **[17]

**Category**	**Relative frequency%**
cardiovascular	21.7
hematologic	10.4
infections	6.7
thoracic	6
lymphatic dysplasia	5.7
placental	5.6
syndromic	4.4
miscellaneous	3.7
urinary tract malformation	2.3
**inborn errors of metabolism**	**1.1**
extrathoracic	0.7
gastrointestinal	0.5
**idiopathic**	**17.8**

According to that study, no definite cause can be found in about 18% of cases of non-immunological hydrops. Chromosomal abnormalities constituted the *chromosomal* group, whereas syndromes, defined as clusters of structural malformations, constituted the *syndromic* group. *Inborn errors of metabolism* were excluded and constituted a separate group including LSD. The authors distinguished between a proven or very likely etiology and a suspected etiology. Regarding the high number (18%) of idiopathic NIHF [17], cases of suspected etiology were included in the idiopathic group.

The relative frequency of the LSDs in the context of NIHF or ascites was 1.4% in a large retrospective series [18]. The relative incidence of metabolic storage diseases as a cause of neonatal ascites is uncertain. A ten-year review of coded diagnoses of congenital ascites at the Hospital for Sick Children, Toronto gave a figure of 11% [19]. In Table 2 the reported incidence of LSD, congenital ascites or IEM respectively in 13 different studies of NIHF is presented.

**Table 2:** Reported incidence of lysosomal storage disorder and inborn errors of metabolism, respectively in 13 different studies on NIHF or congenital ascites

**Author**	**Total number of patients**		**LSD %**
**Gillan**[19]	not defined	10-yr review of coded diagnosis of neonatal ascites	11%
**Burin**[11]	33	NIHF patients (28 pregnancies between 16 and 32 weeks, 5 newborns)	15%
**Machin**[20]	804	Literature review of HF case series (1980-1989)	1%
**Jauniaux**[21]	600	Literature review of NIHF with focus on genetic disorders (1982-1990)	1%
**Groener**[22]	17	NIHF detected by fetal ultrasound	5.9%
**Piraud**[23]	70	AF due to abnormal ultrasound findings (54 cases NIHF)	14.3% - 18%
**Kooper**[24]	75	Pregnancies 14-36 weeks of gestation, AF or cultured amniocytes	5.3% - 8%
**Favre**[16]	79	Fetal ascites of NIHF detected by routine ultrasound during the second or third trimester	8.9%
**Mahoney**[25]	27	Sonographic evaluation 13-34 weeks of gestation with NIHF	3.7%
**Bellini**[17]	6.361	Literature review of NIHF	1.1% (IEM)
**Abrams**[26]	414	NIHF, retrospective review of a large national data set (1996-2005)	1.2% (IEM)
**MacFadden**[27]	90	Review all autopsy cases with HF over an 11- yr period	4.4% (IEM)
**Larroche**[28]	38	NIHF fetuses and newborns with focus on fetal cerebral ultrasounds	7.9% (IEM)

AF: Amnion fluid, IEM: Inborn errors of metabolism, HF: Hydrops fetalis, NIHF: Non Immunological Hydrops fetalis.

Most of the LSDs with hydrops fetalis are autosomal, recessively-inherited disorders with a recurrence risk of 25%. Thus, correct diagnosis is essential for genetic counseling and family planning. There is still some uncertainty about the relative frequency of LSD in NIHF, which may be underestimated. An underlying metabolic disease may be the etiology of a substantial number of these unexplained cases. In recent years it has been recognised that hydrops fetalis may be an extreme presentation of many of the lysosomal storage disorders. It can be hypothesized that lysosomal enzymes and their pathways are bi-functional and hereby different in utero. This may lead to an NIHF which requires further investigation. Usually hydrops fetalis is the most severe phenotype of some LSDs, caused by two deleterious mutations with a complete deficiency of the enzyme or transport protein. In the most severe form of LSD we usually have a good phenotype-genotype correlation, for example, the deletion 84GG always manifests as a collodian-baby in Gaucher disease. On the other hand, Niemann-Pick A does not manifest as an HF even with two knock- out mutations. The two cases reported with NPA and NIHF are an exception and may be due to secondary epigenetic factors [11].

At present, 14 different LSDs are described as being associated with NIHF or congenital ascites [29]. These LSDs associated with NIHF include cases with type 2 Gaucher disease, Sialidosis, Galactosialidosis, ISSD, MPS types IV and VII, GM1 gangliosidosis, I-cell disease, NPA and NPC, Wolman disease, and Farber disease. Cited associations of NIHF with Hurler disease [30] and Multiple sulfatase deficiency [11] seem to be doubtful and there are no cases with a clear description. Thus at present, 12 different LSD may be associated with NIHF (Table 3).

**Table 3:** Lysosomal storage diseases and non- lysosomal inborn errors of metabolism associated with NIHF

**Lysosomal storage diseases**			**Non-lysosomal inborn errors of metabolism**
**transient NIHF/ascites**	**NIHF with prominent ascites**	**NIHF (pericardial and/or pleural effusion, ascites, skin edema)**	
**Mucopolysaccharidosis:**			**Glycogenosis:**
			Type IV (Anderson disease): [31,32]
MPS IVA (this publication)		MPS IVA (Morquio) [33,34]	**Congenital disorder of glycosylation:**
MPS VII (this publication)		MPS VII (Sly): [35-41,14]	CDG Ia [42,43]
			**Peroxismal disorder:**
			Zellweger syndrome [44]
**Sphingolipidosis/Oligosaccharidoses:**			**Fatty acid oxidation defects:**
GM1-Gangliosidosis [14,15] Niemann-Pick C (this publication)	Sialidosis [45-47]	Sialidosis Galactosialidosis [48,49]	Long-chain-hydroxyacyl CoA dehydrogenase deficiency (LCHAD) [50]
	GM1-gangliosidosis [51,15]		
		GM1-gangliosidosis [29]	Primary carnitine deficiency [52]
	Niemann-Pick C [53,54]	Gaucher Type II [55]	**Cholesterol biosynthesis defects:**
		Niemann-Pick A [11,56]	Smith-Lemli-Opitz Syndrome [57]
		Niemann-Pick C [58]	Greenberg syndrome: Hydrops-ectopic calcification moth-eaten skeletal dysplasia [59]
		Farber disease [9,60]	
			Conradi Huenermann: Chondrodysplasia punctata [61] (X linked disorder: male fetus)
**Lysosomal transport defect:**	Infantile sialic acid storage disease [62]	Infantile sialic acid storage disease [63]	
			**Others:**
			Citric-acid cycle defect (Fumarase deficiency) [64,65]
**Others:**	Wolman disease [66]	Mucolipidosis Type II (I-cell disease) [67]	Neonatal hemochromatosis [20]
			Transaldolase deficiency [68]
			S-adenosylhomocysteine hydrolase deficiency [69]
			Congenital erythropoietic porphyria [70]

### LSD in association with hydrops fetalis

There are a variety of descriptions of Type 2 Gaucher disease presenting with hydrops fetalis. In the majority of cases with this severe phenotype including a collodian membrane, glucocerebrosidase activity is absent or severely deficient [23,24,29,55,58,71-74]. Type 2 Gaucher disease is the rarest and most severe type, and untreated patients uniformly die before one year of age [12].

Cases of neonatal Sialidosis presenting as hydrops fetalis or with neonatal ascites have been reported [11,24,29,45,47-49,74-76]. NIHF has been reported in galactosialidosis [48,49,75]. Claeys et al. described a patient who presented prenatally with massive ascites and was diagnosed only after birth as having galactosialidosis [77]. Burin et al. reported hydrops with I-cell disease and NPA [11].

MPS IVA (Morquio disease) is a rare disease, prenatal manifestations of the disease include hydrops fetalis [33,34]. MPS VII has also been recognized as a cause of NIHF, which is actually the most common presentation of the disease [35,73]. However, there is great variability in the associated clinical and biochemical manifestations [36]. The reported case I of this publication seems to emphasize this variability. A preterm infant of 29 weeks of gestation with Farber disease and severe hydrops fetalis is reported [9,60].

### Congenital ascites in association with LSD

There have been at least five descriptions of cases with NIHF or congenital ascites, either transient or persistent, as the presenting symptom of GM1 gangliosidosis [15,51,74]. As in case IV of this publication, Maconochie et al. described a patient with severe congenital ascites who had been diagnosed with NPC [53], and Meizner et al. reported a case of NIHF in which NPC was diagnosed by electron microscopy [58]. Although Wolman disease is usually accompanied by mild ascites, Uno et al. [78] and Ben-Haroush et al. [66] each described a case with isolated massive milky ascetic fluid. ISSD have been described in patients with massive fetal ascites [62]. Daneman et al. looked at four newborns with LSD, in whom the dominant presenting clinical feature was ascites. The diseases included Gaucher disease, GM I gangliosidosis, Sialidosis, and ISSD. Abdominal distention due to ascites and hepatosplenomegaly and hypoplastic lungs were seen in all patients [79].

### Transient NIHF in association with LSD

In the literature two cases of transient NIHF in association with LSD are described. The first case occurred as early as 28 weeks of gestation and otherwise uneventful pregnancy of a non-consanguineous couple. At birth he had mild dysmorphic features and mild hepatosplenomegaly. After three days, neurological deterioration was observed and successively the diagnosis of GM1 gangliosidosis was made [14]. The second case of transient hydrops reported (week 28 of gestation) was also a GM1 gangliosidosis and diagnosed on the basis of pathological placental findings after birth [15]. The patients reported in this publication with *transient* hydrops in association with MPS IVA, MPS VII and NPC are the first described cases in the literature.

### Suggested diagnostic approach

Staretz-Chacham and colleagues proposed an algorithm for the clinical evaluation of a fetus or newborn with NIHF [12]. Where there is facial dysmorphism, irregularity of the epiphyses, and coarse trabeculations of the long bones in the presence of congenital ascites, the index of suspected storage disease is even greater [12].

We herewith suggest a diagnostic approach to the fetus with NIHF with regard to suspected LSD (Table 4). It may be considered that before week 18 of gestation, urine production of the fetus is limited, bringing the risk that a lysosomal diagnosis would be missed at the metabolite level (amnion fluid). The combination of measurements at the metabolite and the enzyme levels will allow a diagnostic laboratory to pick up the most frequent LSD known to be associated with NIHF [24].

**Table 4:** Diagnostic approach for NIHF and suspected lysosomal storage disease

**Examination of the fetus with NIHF/ascites in utero**	**Examination of the dead fetus with NIHF**	**Examination of the live neonate with NIHF**
		**Screening**
**1. Amniotic fluid** GAG-electrophoresis*, lysosomal enzymes elevated in ML II	**1.Postnatal autopsy** should be discussed in every individual fetus	**1. Placental tissue** for histological analysis. Culture of this tissue is also possible (cell lines often show early senescence)
	-Babygram	If possible elctronmicroscopy for abnormal lysosomal shingolipid storage- NPC)
	-Photo-documentation	
**2. Amnion cells/Chorionic villi:** enzyme measurements of ß-Glucocerebrosidase (Gaucher disease), N-Acetyl-Glukosamin-6-Sulfatsulfatase (MPS IVA), ß-Glucoronidase (MPS VII), Neuraminidase and ß-Galactosidase (Galactosialidosis, GM1 Gangliosidosis and Sialidosis). Ceramidase (Farber disease), Acid lipase (Wolman disease), Sphingomyelinase (NPA)		**2. Urine** for oligosaccharides and GAG-electrophoresis
	**2. Skin biopsy:** DNA isolation for genetics and cultivated cells for biochemical tests (enzyme analysis)	**3. Screening test in Plasma/Serum:** I-cell screen^**^, chitotriosidase
		**4. Blood smear**: vacuolated granulocytes and Adler granulation (GM1-gangliosidosis, Galactosialidosis, Sialidosis, ISSD, MPS VII)
	If possible electronmicroscopy	
	**3. Tissue sample** (unfixed) e.g. liver, spleen, heart, and muscle for histopathological and histochemical examinations.	
		**5:** Considering **bone marrow aspiration** to look for foam cells, Gaucher cells and other storage histiocytes)
	**4. Placental tissue** for histological analysis. Culture of this tissue is also possible (cell lines often show early senescence)	
		**6. Skeletal-radiography:** dysostosis multiplex, pathological fractures (ML II)
	If possible elctronmicroscopy for abnormal lysosomal shingolipid storage- NPC)	**Diagnosis**
	**5. Fetal urine** (if available) for Glycosaminoglycans or Oligosaccharides	
**3. Genetic analysis:** amnion cells or chorion villi cells		**7. Skin biopsy:** (best with eccrine glands) DNA isolation for genetics and cultivated cells for biochemical tests (enzyme analysis), Filipin test*** for NPC, abnormal lysosomal free sialic acid storage (ISSD)
		If possible electronmicroscopy
		**8. Leucocytes and plasma (also possible in fibroblasts):** measuring lysosomal enzymes of ß-Glucocerebrosidase (Gaucher), N-Acetyl-Glukosamin-6-Sulfatsulfatase (MPS IVA), ß-Glucoronidase (MPS VII), Neuraminidase and ß-Galactosidase (Galactosialidosis, GM1 Gangliosidosis and Sialidosis) Ceramidase (Farber disease). Acid lipase (Wolman disease), Sphingomyelinase (NPA).
		**9. EDTA-blood** for DNA isolation

* GAG-electrophoresis: electrophoresis of glycosaminoglycans, ** I-cell screen: lysosomal enzyme activity in serum more than 10 times the reference range. Diagnosis in fibroblasts or amnion cells: same lysosomal enzymes deficient. ***Filipin test: reaction of impaired cholesterol esterification in cultured cells with fluorescent filipin in NPC.

It is very important to examine the placenta carefully in cases where hydrops or ascites are present at birth or detected by ultrasound, especially in the transient form. Placental histology can serve as an early diagnostic clue for a number of storage diseases, including GM1 gangliosidosis, MPS VII, ISSD, Gaucher disease, galactosialidosis and Fabry disease. The presence of highly vacuolated cells or cells demonstrating storage should be followed up with enzymatic testing in patients [12]. Even if a family does not agree to autopsy, placental examination may be done. In some instances, examination of the placenta of a grossly macerated fetus may be more informative than that of the fetus. Unfortunately this was not done in the patients reported in this publication.

### Family history

Because most LSDs are inherited in an autosomal recessive manner we have to consider the importance of possible consanguinity. Often, reported patients had an antecedent sibling who was also affected but not diagnosed. This was true in the family history of case III of this publication. For this reason, in cases of familial NIHF, one should consider the LSDs or other IEM.

Nelson et al. described a case of MPS VII in consanguineous parents who had three previous unsuccessful pregnancies because of recurrent stillbirths [37]. Landau et al. described a family in which fetal hydrops occurred in four pregnancies, and the diagnosis of galactosialidosis was only made after the birth of the fourth affected child [48]. Gillan reported eight cases of neonatal ascites associated with different types of LSD: sialidosis, ISSD, GM1 gangliosidosis, and Gaucher disease. In each case there was a history of sibling perinatal death resulting from the disease [19]. Manning et al. described the case of a twelfth pregnancy of a woman who had four first-trimester miscarriages and a previous child who died at four weeks of age. Ultimately, the diagnosis of NPC was made [54]. In the four patients reported by Daneman, there was a history of perinatal death due to the same disease in a sibling. The diagnosis of LSD was missed at autopsy in each of these siblings, underlining the lack of awareness of LSD as a cause for neonatal ascites [79].

## Conclusion

From the cases reported in the literature it can be concluded that the diagnosis of a high proportion of NIHF, and especially of LSD are missed. As already suggested by Kooper and colleagues [24], this paper emphasizes the fact that the incidence of LSD may be significantly higher in NIHF than the estimated 1% reported in previous studies. However, the higher incidence could be due to an ascertainment bias, again due to referral to a specialized center for metabolic diseases. But, as also stated by some authors, the given low incidences, in contrast, could be the result of incomplete investigation of NIHF [19,24].

Transient forms of NIHF and/or ascites in association with LSD as described in this publication in MPS IVA, MPS VII and NPC for the first time must be considered in the differential diagnostic of LSD.

Extensive and thorough investigation for the etiology of NIHF, congenital ascites and transient hydrops as proposed by the diagnostic approach in this publication seems to be obligatory. Enzymatic studies in chorionic villous sample or amniotic cultured cells, once the most common conditions associated with fetal ascitis or hydrops have been ruled out, are important.

A prospective registration and complete examination of all cases of NIHF appears to be mandatory.

## Abbreviations

AF: Amniotic fluid; HF: Hydrops fetalis; GAG: Glycosaminoglycans; IEM: Inborn errors of metabolism; ISSD: Infantile Sialic Acid Storage Disease; LSD: Lysosomal storage disease; ML II: Mucolipidosis (I-cell disease); MPS: Mucopolysaccharidosis; NIHF: Non-immunological hydrops fetalis; NPA: Niemann Pick A disease; NPC: Niemann Pick C disease.

## Competing interests

E. Mengel and M. Beck received research grants, consulting fees, travel grants and speaking fees from Genzyme, Shire, Actelion and Biomarin.

## Authors’ contributions

CW, EM, MB and EM have conceived the initial concept. All authors were involved in treating patients and collecting data. CW had full access to all of the study data and takes responsibility for their integrity and the accuracy of the data analysis. CW drafted the first proposed manuscript. EM and EM complemented the initial drafts and expert contributed input, which was then corrected and approved by all authors. All authors read and approved the final manuscript.
